# An improved Fourier-Transform Infrared Spectroscopy combined with partial least squares regression for rapid quantification of total aflatoxins in commercial chicken feeds and food grains

**DOI:** 10.5455/javar.2022.i624

**Published:** 2022-09-30

**Authors:** Bahauddeen Salisu, Siti Marwanis Anua, Wan Ishak Wan Rosli, Nurzafirah Mazlan

**Affiliations:** 1 School of Health Sciences, Health Campus Universiti Sains Malaysia, Kelantan, Malaysia; 2 Department of Microbiology, Umaru Musa Yaradua University, Katsina, Nigeria; 3 Borneo Marine Research Institute, Universiti Malaysia Sabah, Sabah, Malaysia

**Keywords:** Aflatoxin contamination, dietary%20exposure, spectroscopy, food safety, hepatocellular carcinoma, PLSR model

## Abstract

**Objective:**

This study aims to develop and validate an Attenuated Total Reflectance–Fourier Transform Infrared Spectroscopy (ATR-FTIR) spectroscopic technique combined with a partial least squares regression (PLSR) model for rapid quantification and monitoring of aflatoxins in chicken feeds and food grains.

**Materials and Methods:**

A model of ATR-FTIR-PLSR was developed using ATR-FTIR spectra of mixed aflatoxin standards in 100% acetonitrile (112 samples) and 75% methanol (112 samples), validated by testing its prediction on 125 feed/food samples spiked with variable concentrations of aflatoxins, and applied to screen 660 samples of commercial chicken feeds and food grains from Nigerian and Malaysian markets for total aflatoxins, for which the dietary exposure risks to aflatoxins (DERA) and associated hepatocellular carcinoma (HCC) risks were evaluated for both countries.

**Results:**

The ATR-FTIR-PLSR model demonstrated excellent prediction power [*R*^2^ = 99.59%, p = 0.001, root mean square error of calibration (RMSEC) = 1.69, RMSE p = 1.98, bias = −0.26], sensitivity (limit of quantitation and limit of the method < 5.0 ng/gm), precision (coefficient of variation = 0.97–1.72), and accuracy (% recovery of 88%–106%) in all the spiked samples. The model’s prediction was statistically reliable (*R*^2^ = 99.8%, p < 0.05) when compared with a high-performance liquid chromatography method. Levels of aflatoxins in the commercial samples signify high DERA (0.92–138.2 ng of aflatoxins/kg BW/day) and HCC risk (1.07%–159.91% of HCC/100,000 people/year) in the exposed populations.

**Conclusions:**

Results feature the conceivable implementation of the proposed ATR-FTIR-PLSR model for rapid, accurate determination and monitoring of aflatoxins in commercial chicken feeds and food grains; and the need to strengthen aflatoxin control/prevention strategies in the study populations.

## Introduction

Different mycotoxigenic fungi can contaminate several crops at pre-harvesting, harvesting, postharvest handling, and transportation stages, or when the farm products are stored improperly by farmers and/or other stakeholders [1–3], leading to spoilage and mycotoxin contamination, which consequently leads to severe economic loss and the manifestation of both chronic and acute forms of mycotoxicosis in exposed humans and animals. This has led to the continuous demand for rapid, reliable, and sensitive techniques for accurately determining mycotoxin contamination in feeds and foods.

Aflatoxins have been described as the most toxic mycotoxins that commonly contaminate stored foods and feeds [4–7], leading to chronic or acute aflatoxicosis (hepatotoxicity, teratogenicity, nephrotoxicity, enterotoxicity, mutagenicity, neurotoxicity, and often death) in exposed subjects. Research has shown that aflatoxins decompose at 200^o^C–300^o^C; hence, they are not denatured by pasteurization or most industrial or home food/feed processing processes. Humans become exposed to aflatoxins directly through oral ingestion of contaminated food [8–10] or indirectly through milk consumption, eggs, and/or meat of animals that have fed on aflatoxin-contaminated feed. Occupational exposure also occurs via dermal routes or inhaling dust generated while handling and/or processing aflatoxin-contaminated grains and feeds [11,12]. Thus, food grains and feed represent a primary source of human exposure to aflatoxins. Hence, the need for rapid on-the-spot methods of quantifying aflatoxins in food grains and feeds is of paramount importance.

Today, most of the regularly used procedures [thin layer chromatography, high-performance liquid chromatography (HPLC), enzyme-linked immunosorbent assay (ELISA), etc.] to detect aflatoxins are tedious, costly, and require sufficient mastery or expertise [13]. On the other hand, spectroscopic techniques, for example, Attenuated Total Reflectance–Fourier Transform Infrared Spectroscopy (ATR-FTIR), FTIR, Raman spectroscopy, Fourier transform–near-infrared reflectance (FT-NIR), NIR, have many fascinating features, for example, simple operation, cost-effectiveness (no consumables required), non-destructive property, the logical speed with little or no sample preparation stage, and the ability to deliver a lot of subjective and quantitatively reliable data relating to the compound structure of aflatoxin particles from a tiny sample portion at a single scan [14,15]. In general, the most precise routine techniques (HPLC and ELISA) for aflatoxin analysis are slower than the spectroscopic methods [16]. In addition, the chemical and immunologic methods require more costly chemicals and instruments compared to the spectroscopic methods. Hence, spectral detection is simpler and more economical.

Spectroscopic techniques have been used to determine aflatoxin and fungal contamination of food grains and poultry feeds [13,15,17–23]. Of these spectroscopic techniques, ATR-FTIR offers additional advantages in aflatoxin analysis due to its non-sample preparation requirement and the ability to allow full mid-infrared (MIR) range use with no limitations caused by saturation impacts of the OH vibrational groups [24]. In addition, it can detect important functional groups (e.g., C–O, and C=O) that are not detected by other spectroscopic techniques such as NIR [25]. In fact, ATR-FTIR spectra are easier to interpret than NIR and exhibit higher specificity and selectivity [26–28]. Hence, it is the most desirable for qualitative and quantitative analyses [16].

Despite the advantages of ATR-FTIR over other spectroscopic methods, studies reporting its application to determine aflatoxins in staples are inadequate. The technique has been used to quantify individual aflatoxins in peanut cake [17] and brown rice [29], achieving an *R*^2^ value above 0.97 and 0.92, respectively. However, both studies were on “individual aflatoxins,” not the evaluation of total aflatoxins. Besides, the former study was on “peanut cake,” not “peanut grains.” In another study [18], an FTIR-partial least squares regression (PLSR) model was developed from eight concentrations of aflatoxin standards (0–70 ppb) and applied to quantify total aflatoxins in eight broiler feed samples. However, it is easy to note that the reported PLSR model needs improvements or further evaluation because it was developed from a few samples (8 samples only). In contrast, the standard PLSR model for quantification purposes should be developed from at least 100 samples [30]. Some other literature also reports MIR application to efficiently discriminate atoxigenic and toxigenic fungi-and aflatoxin contamination of dried fruits and nuts [1,22,23,31]. But there aren’t many studies that show how ATR-FTIR-PLSR is used to measure the total amount of aflatoxins in foods and feeds.

With the preceding in mind, this study was conducted to (1) develop and validate a simple, cost-and time-effective model of PLSR integrated ATR-FTIR spectroscopy for the quantification of total aflatoxins in commercial chicken feeds and food grains, using specific MIR regions (rather than the whole spectrum used by most previous studies), (2) correlate the aflatoxin levels determined by the ATR-FTIR-PLSR model with those obtained by the HPLC method, (3) determine the versatility of the developed ATR-FTIR-PLSR model by applying it for the quantification of total aflatoxins in 660 samples of commercial chicken feeds and food grains from some Nigerian and Malaysian open markets, and (4) evaluate the aflatoxin exposure/hepatocellular carcinoma (HCC) risks in the exposed populations of both countries.

## Materials and Methods

### Sample collection and extraction

A total of 660 commercial samples of chicken feed and food grains were obtained from some Malaysian and Nigerian open markets. For the analysis, a total of 220 composite samples were prepared from the 660 samples. Each composite sample was made from three samples of the same kind which were bought from different vendors or sellers in the same market. The total composite samples made from samples in each sampling environment are as follows:

1. Forty-eight composite samples of chicken feed were purchased at open marketplaces in Katsina state, Nigeria;
2. Eighty-four composite samples of food grains consisting of 21 composite samples each of rice, wheat, maize, and peanuts from open marketplaces in Katsina state, Nigeria;
3. Forty-four composite samples of chicken feed from poultry shops in Kelantan state, Malaysia; and
4. Forty-four composite samples of food grain consisting of 11 composite samples each of rice, wheat, maize, and peanuts from open marketplaces in Kelantan state, Malaysia.

The samples were extracted based on the Quick, Easy, Cheap, Effective, Rugged, and Safe method [32] combined with a double extraction protocol of aflatoxins [33] with some modifications to achieve satisfactory yield/recovery of the aflatoxins. Each sample was grounded into crystalline form, and subsequently, an aliquot of each grounded sample (20 gm) was transferred into a separate conical flask and extracted for 1 h by percolation using 100 ml of extraction solvent (methanol + water, 3:1, v/v) + 5% (5 gm) of salt mixture (sodium chloride + magnesium sulfate, 1:4, w/w). The extraction was carried out on a Scilogex LCD digital orbital shaker (Westwood, USA) at 100 rpm. Next, a high-speed blender was used to blend the mixture for 2 min, then allowed to settle for 1 h to form two separate layers (aqueous and organic layers). The organic layer was carefully decanted and filtered using Smith 102 Qualitative filter paper. Using the same protocol, the residue was re-extracted three more times with 50 ml of the extraction solvent, each time filtering out the organic layer. The total organic layer filtrate obtained from each sample was evaporated in an evaporating oven at 35^o^C, after which the resulting extract was weighed and resuspended in 2 ml of 75% methanol (75% MeOH) in an amber vial and stored at 4^o^C in a dark, cold room until needed for downstream application. The percentage yield/recovery of each aflatoxin based on the current extraction protocol was assessed (using blank samples spiked with 5, 10, and 15 ng/gm of the mixed aflatoxins, extracted and analyzed by HPLC) to ensure it is within the recommended range (70%–110%) for aflatoxins as established by the Commission Regulation of the European Communities [34] before adopting the extraction method.

### Preparation of spiked samples

The spiking of the samples with the desired concentrations of the mixed aflatoxin standard solution was carried out using the good quality chicken feed, rice, wheat, maize, and peanut. The samples were pre-screened using HPLC to ensure they had no traceable level of aflatoxins. The samples were fortified with a working standard solution containing 200 ng/ml of each of the four aflatoxins using Equation (1) below [35].


$$\mathrm{Aflatoxinspiketospike}\;(\mathrm{ml})\;=\;\frac{\mathrm{Sample}\;\mathrm{weight}\;(\mathrm{gm})\;\times \;\mathrm{Desired}\;\mathrm{spiking}\;\mathrm{level}\;\left(\frac{\mathrm{ng}}{\mathrm{gm}}\right)}{\mathrm{Concentration}\;\mathrm{of}\;\mathrm{aflatoxin}\;\mathrm{standard}\;\left(\frac{\mathrm{ng}}{\mathrm{gm}}\right)}\;\;\;(1)$$

Each spiked sample was thoroughly mixed to ensure the even homogenization of the mixed aflatoxin solution. Next, the spiked samples were stored at room temperature for 24 h to allow solvent evaporation and sample-analyte equilibration to mimic the natural contamination process. After that, the samples were ground into crystalline form and extracted using the double extraction method described for the commercial samples above.

### Preparation of mixed aflatoxins‘ standard solution

Five ml of a certified mix of four standard aflatoxin solutions containing an equal ratio of the four aflatoxins [Aflatoxin B1 (AFB1), Aflatoxin B2 (AFB2), Aflatoxin G1 (AFG1), and Aflatoxin G2 (AFG2)] was supplied by Pribolab, China (STD#1089). Two sets of dilutions of the mixed aflatoxin standard solution were prepared in (75% MeOH) (first set) and 100% HPLC-grade acetonitrile (ACN) (A998-4, Fisher Scientific Malaysia) solvent (second set). Each set contains 16 concentration levels (0, 1, 5, 10, 15, 20, 25, 30, 35, 40, 45, 50, 60, 70, 80, and 90 ng/ml) of the mixed aflatoxin standard solution. Each concentration was prepared in 7 different vials, making a total of 112 vials for each set (first set and second set).

### Analysis of aflatoxins by HPLC

The HPLC analysis was carried out as described in the aflatoxin standard‘s certificate of analysis (Pribolab, China) with few modifications to achieve optimal separation of the four aflatoxins. The HPLC was performed using Nexera HPLC with fluorescence detection (LC-20AD Shimadzu, Japan). The samples were filtered using a micro-syringe filter (0.2 µm, Thermo Fisher Scientific, Malaysia) and loaded into the HPLC sample tray. The separation was carried out in a 5 m Agilent Zorbax column [HC-C18(2) (Agilent Technologies, Netherlands)]. Optimal separation of the four aflatoxins was achieved using a mobile phase consisting of ACN + methanol + water (15:25:60 v/v) at an injection volume of 10 µl and a flow rate of 0.50 ml/min for 25 min with a column oven temperature of 40^o^C, a 360 and 450 nm excitation wavelength and emission wavelength, respectively. A calibration curve for each aflatoxin was generated using a set of concentrations (1.0, 2.0, 4.0, 6.0, and 8.0 ng/ml) of the mixed aflatoxin standard solution. By analyzing a set of concentrations of food grains and poultry feed spiked with known amounts (4, 8, and 16 ng/gm) of the mixed aflatoxin standard solution, the HPLC method was further optimized for specificity, precision, and accuracy.

### Analysis of aflatoxin by ATR-FTIR spectroscopy

The analysis was performed using a Bruker Tensor 27–Fourier Transformed Infrared spectrometer (Bruker, MA). All the samples were equilibrated at 25^o^C for 1 h before the analysis. An aliquot of 20 µl of each sample was applied directly to the ATR diamond (Lumos Bruker, USA) and scanned between the MIR region of 4,000 cm^−1^ and 600 cm^−1^ for 32 scans at a resolution of 4 cm^−1^, smoothed at polynomial order 3.0, and averaged as a single spectrum. Each sample was analyzed in triplicate to minimize sampling error, averaged as a single spectrum, and used for subsequent analyses. The ATR crystal interface was adequately cleaned (3 times with 75% MeOH) and dried between successive sample applications. A background spectrum was also recorded before each sample measurement and subtracted from the sample‘s spectrum.

### ATR-FTIR-PLSR model development and validation

The most common multivariate calibration method for data analysis and developing quantitative models is partial least squares (PLS) regression [36–38]. In this study, following the ATR-FTIR spectra acquisition of the 112 samples of the mix aflatoxin standard for both ACN and 75% MeOH sets, specific spectra regions, common in both groups, that describe most of the aflatoxin characteristics were selected and used for developing the PLSR model. The chosen frequency regions were evaluated for linear proportionality between the absorbance (responses) in the whole selected spectra region and the analyte concentrations (aflatoxins) [39]. The PLSR calibration model was developed with cross-validation in which leave-one-out was used to achieve internal validation of the data sets [40]. Next, the PLSR model at the common wavenumber region in both ACN and (75% MeOH) groups that gave the highest correlation between the analyte concentrations and the absorbance responses within the acceptable bias limit (+2δ and −2δ) was chosen for further validation. The selected frequency region was chosen based on having the lowest value of root mean square error of calibration (RMSEC) and the highest value of the coefficient of determination (*R*^2^) in reference to Rohman et al. [39].

The developed PLSR calibration model was further validated for accuracy, precision, and specificity by testing its prediction on an independent set of 125 samples consisting of 25 samples each of chicken feed, rice, wheat, maize, and peanut in reference to Luna and de Gois [40]. These 125 samples (10 gm each) were fortified with various concentrations of mixed aflatoxin standard solution at five different spiking levels (0, 2, 4, 8, 16, and 64 ng/gm) by the standard addition method in reference to Sherazi et al. [18]. All the samples were analyzed using the ATR-FTIR at room temperature, and the obtained spectra were used to evaluate the prediction power of the ATR-FTIR-PLSR model based on the *R*^2^ values. The root means a square error of prediction (RMSEP) [41].

Furthermore, the performance of the ATR-FTIR-PLSR model was compared with that of the optimized HPLC method on the same set of concentrations (0–90 ng/gm) of the mixed aflatoxin standard solution, and the spiked samples by fit regression between HPLC measured concentrations versus the ATR-FTIR-PLSR predicted concentrations.

### Determination of total aflatoxins in the commercial chicken feeds and food grains samples

The validated ATR-FTIR-PLSR model was used to determine the total aflatoxins in the extracts of the 220 chicken feed and food grain samples from Malaysia and Nigeria. Each of the extracts was dissolved in 2 ml of (75% MeOH) , vortexed, and equilibrated at 25^o^C for 1 h before FTIR analysis. The ATR-FTIR spectrum of each sample was then analyzed using the ATR-FTIR-PLSR model for total aflatoxin prediction.

### Exposure risk assessment of Aflatoxins

The mean and range of aflatoxins in the contaminated food grains were used to determine the estimated exposure to aflatoxins in ng/kg BW/year in Malaysia and Nigeria. Subsequently, the estimated incidence rate of HCC per 100,000 population per year was calculated for both countries.

### Data analysis

Minitab version 18 (Minitab LLC, USA), Spectragryph optical spectroscopy software version 1.2.14/2020 [42], and Microsoft Excel were used to process and analyze the spectral data. The level of contamination in the samples was summarized using descriptive statistics. The level of agreement between the HPLC and the ATR-FTIR measurements of aflatoxin concentrations was determined using Bland-Altman‘s test. Also, a one-way analysis of variance was used to determine if the levels of aflatoxins in the sample categories differed.

### Raw data availability

The present study’s raw data is available in Mendeley with the following reserve doi: http://dx.doi.org/10.17632/y7fzbxpzd5.1

## Results and Discussion

### HPLC analysis

The HPLC optimization result on the mixed aflatoxin standard solution and spiked chicken feeds and food grain samples showed no interference between the aflatoxin peaks and the sample matrix, demonstrating the method‘s excellent sensitivity and specificity. With a *R*^2^ values of 0.9991, 0.9999, 0.9997, and 0.9994 for AFB1, AFB2, AFG1, and AFG2, respectively, the technique also demonstrated good linearity throughout a concentration range of 1.0–8.0 ng/ml. Figure 1 displays a comparison of the chromatograms demonstrating the linearity of the HPLC technique in the spiked maize sample. The method‘s accuracy was determined by analyzing three samples of each of the chicken feed and food grains spiked with 4.0, 8.0, and 16.0 ng/gm of the mixed aflatoxin standard by the standard addition method [43] in triplicate. The percentage recoveries of the aflatoxins in all the spiked samples ranged from 97% to 105%, implying good accuracy and precision of the method. So, the new ATR-FTIR-PLSR method was used to compare the total amount of aflatoxins in the samples that had been tampered with.

### ATR-FTIR spectra measurements

In this study, aflatoxin standard solutions were prepared in two different solvents (75% MeOH and ACN) to increase the accuracy in identifying the aflatoxin peaks for model development. The ATR-FTIR spectra of the various concentration levels of the mixed aflatoxin standards prepared in 75% MeOH and ACN solvents are shown in Figure 2. Structurally, aflatoxins contained six different functional groups (-CH3, -C-H, C = C, = CH, -C = O, and = C-O-C) belonging to aromatic ethers, phenyls, and ketones.

As depicted in Figure 2, the various spectra peaks representing the aflatoxins‘ functional groups are labeled with the respective functional groups‘ names. The -CH3 group has one peak in 75% MeOH (1,450 cm^−1^) and two peaks in ACN solvent (1,450 and 1,375 cm^−1^). This little disparity between the two solvents is possible because FTIR is a highly sensitive method that can discriminate any small change between materials of the same kind [23,39,44,45], since the vibrational frequency of compounds can be affected by the presence of certain compounds, making it possible for a single compound to have many vibrational frequencies in a different medium [46]. Same observation was also noted in the absorption pattern of the -C-H group (having absorption at 3,002 and 2,826 cm^−1^ in ACN, 2,834 cm^−1^ in 75% MeOH, and 2,970 cm^−1^ in both solvents), = CH group (having absorption at 3,002 cm^−1^ in ACN and 2,970 cm^−1^ in both solvents), C = C groups (having absorption at 3,002 cm^−1^ and 1,635 cm^−1^ in ACN, 1,620 cm^−1^ in 75% MeOH, and 2,970 cm^−1^ in both solvents), and C = O group with absorption at 1,739 cm^−1^ in ACN and 1,749 cm^−1^ in 75% MeOH. Only the ether group ( = C-O-C) has a common peak (1,035 cm^−1^) in both solvents. However, no significant difference was observed between the average absorbance or peak area of the aflatoxin peaks in 75% MeOH and the corresponding peaks in ACN (*p* > 0.05). Overall, our result is comparable with many previous studies that report FTIR spectra of aflatoxins [1,17,18].

**Figure 1.**
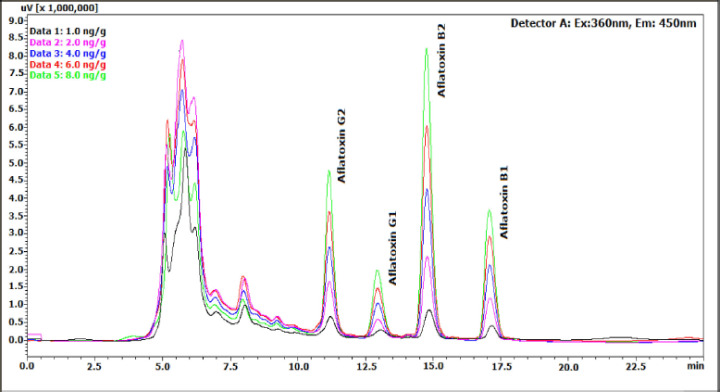
Chromatogram data comparison demonstrating the HPLC method‘s linearity in spiked maize samples across the concentration range of 1.0–8.0 ng/gm.

### ATR-FTIR-PLSR model development and validation

One of the alluring features of FTIR is its ability to collect accurate information on the types of functional groups and bonds present in compounds (based on the vibrations produced when the compound interacts with infrared light/radiation) and present them in the form of spectral fingerprints peculiar to the compound being analyzed, with variable peak intensities that correlate with the concentrations of the respective functional groups present in the analyte. This means that the concentration of the analyte can be predicted using statistical software based on the intensity of the peaks in the spectrum [16].

As depicted in Figure 2, the FTIR peak 2,970 cm^−1^ (for aromatic C = C, -C-H, and = CH ), 1,450 cm^−1^ (for the -CH3 group), and 1,035 cm^−1^ (for the ether group = C-O-C) are the three aflatoxin peaks that have common wavenumbers in both solvents. So, these frequency regions were checked for linear proportionality between the absorbance (responses) in the whole selected spectral region and the concentrations of the analyte (aflatoxins) using PLSR in Minitab software version 18 (Minitab LLC, USA) to see if they could be used to make the PLSR prediction model.

The PLSR model selection result is summarized in Table 1 and depicted in supplementary Figures 1S and 2S. The result showed that all the three spectral regions from both solvents could predict aflatoxin concentration, having *R*^2^ values above 95%, RMSEC of less than 10.0, and *p*-values of 0.001–0.003, which is less than the significant value of 5% (0.05). The only exception is the IR region of the aflatoxins at wavenumber 1,461–1,440 cm^−1^, which has an *R*^2^ of < 72% and an RMSEC of >15.00 in both solvents. Generally, the level of agreement between the acceptable valid values and the estimated values for the calibration samples used to derive PLSR model parameters is described by the *R*^2^ and RMSEC values [40]. The *R*^2^ values above 95% indicate a good calibration model [37,47,48]. In the present study, therefore, the best IR region showing the highest correlation between the absorbance responses and the respective aflatoxins’ concentration in both solvents is the 1,062–1,000 cm^−1^ region, which has the highest absorbance intensity at 1,035 cm^−1^ (Table 1). This frequency region has the highest *R*^2^ value (>99%), and lowest RMSEC (< 2.5) in both solvents, producing the excellent PLSR response plots (Fig. 3) of the FTIR predicted concentration versus the actual concentration of aflatoxins in 75% MeOH and ACN, respectively. The results showed that using the whole MIR or group of frequency regions in the MIR to quantify aflatoxins by PLSR will result in low sensitivity. As shown in Table 1, the PLSRof the combined three best frequency regions (2,970 +1,450 + 1,035 cm^−1^) has lower *R*^2^ and higher RMSEC in both solvents than the chosen 1,035 cm^−1^ region.

The relationship between the absorbance responses and the aflatoxin standards concentration at the optimized frequency region (1,062–1,000 cm^−1^) was further tested; both the 75% MeOH and ACN spectra were selected and integrated at a common baseline using Spectragryph spectroscopy software version 1.2.14 [42], as shown in Figure 4 and Supplementary Figure 3S. The spectra‘s peak areas and average absorbances showed a good linear correlation with the respective aflatoxin concentration, producing the regression equations and good *R*^2^ values shown in Table 2. The ATR-FTIR spectra of aflatoxins in 75% MeOH have slightly higher *R*^2^ values (*R*^2^ > 90%) than the spectra of aflatoxins in ACN; hence, the PLSR model of aflatoxins‘ spectra in 75% MeOH was selected for further validation. This observation further showed that methanol–water is the best solvent for quantifying aflatoxins in foods and feeds by ATR-FTIR. Similarly, Sherazi et al. [18] also made a comparable observation. They obtained a high *R*^2^ when quantifying aflatoxins in poultry feeds using FTIR.

For the external validation, the chosen ATR-FTIR-PLSR model was subjected to evaluation using 125 independent sets of chicken feeds, rice, wheat, maize, and peanut samples fortified with 0–64 ng/gm of the mixed aflatoxins by the standard addition method to validate its specificity, precision, and accuracy of prediction/prediction power in the quantification of total aflatoxins in chicken feeds and food grains. A statistically good prediction power was obtained in all five categories of spiked samples, as evidenced by *p*-values of less than 0.05, *R*^2^ values above 95%, RMSEP below 10, high precision [coefficient of variation (CV), values between 0.97 and 1.72], and high accuracy (percentage recovery values between 88% and 106%). Both the *R*^2^ and RMSEP values indicate the high sensitivity and precision of the developed model, as ascertained by several studies that used PLSR for the quantification of various analytes [16,26–28,36–38,40]. In addition, the recoveries of the aflatoxins in the spiked samples are within the acceptable limits defined by the European Union [49].

As for the method’s sensitivity, in all the spiked samples, the aflatoxin concentration at 2.0 ng/gm was predicted reasonably accurate. Therefore, the detection limit of the method (LoD) is suggested at 2.0 ng/gm. However, the concentration of mixed aflatoxins at 4.0 ng/gm was much reliably detected in all the spiked samples. Hence, it is recommended as the method’s limit of quantitation (LoQ) for total aflatoxins in the chicken feeds and food grains. These observations have been corroborated by Sheraziet al. [18], who obtained the LoD of 1.5 ng/gm for total aflatoxins in poultry feed using FTIR.

**Figure 2.**
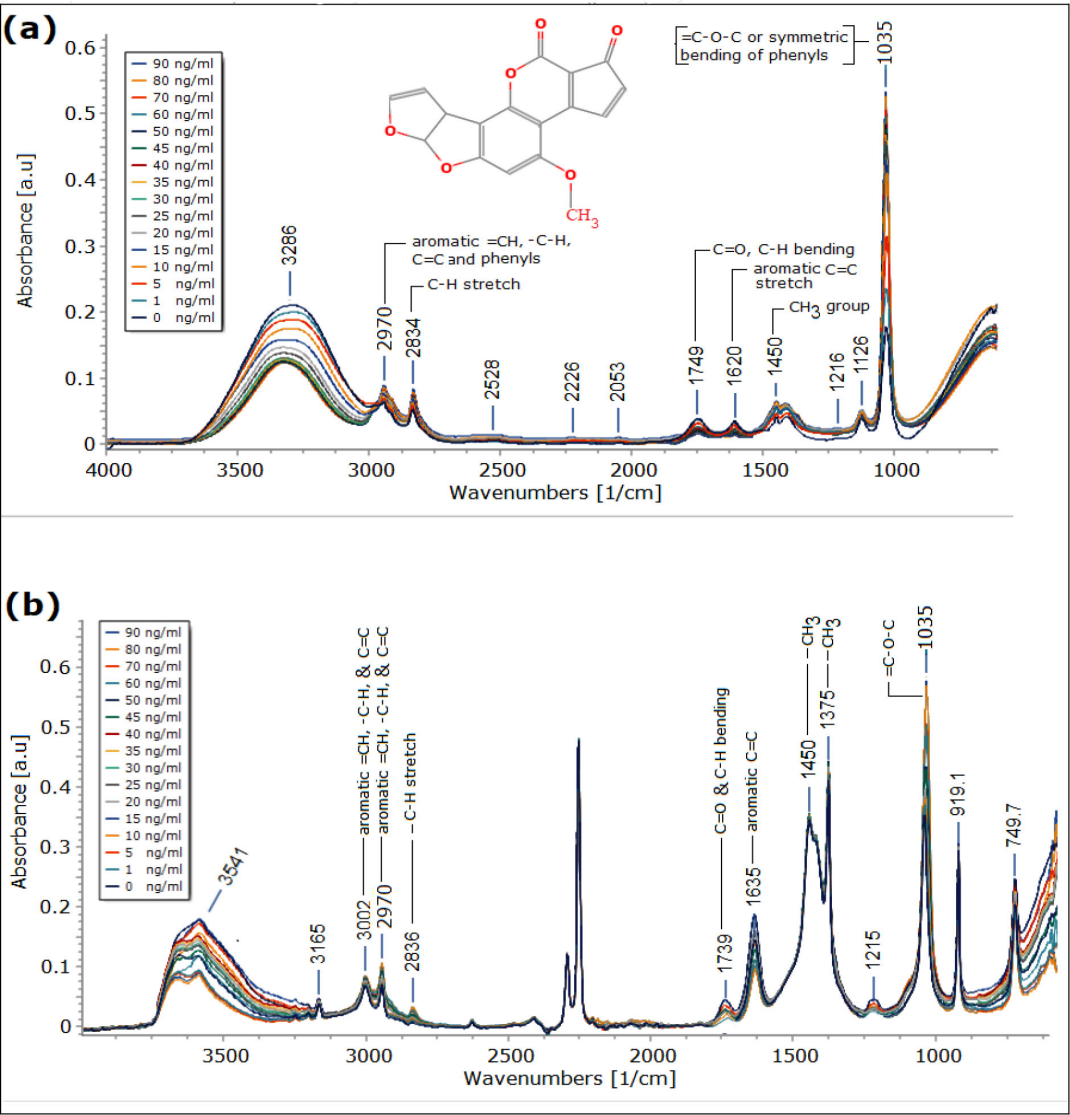
The chemical structure of aflatoxin (AFB1) and the MIR ATR-FTIR spectra of the various dilution groups (0–90 ng/ml) of standard aflatoxin mixture in (a) (75% MeOH) and (b) ACN solvent. The aflatoxin peaks common in both solvents are labeled with the respective functional groups they signify.

Furthermore, when the performance of the ATR-FTIR-PLSR model was compared with that of the optimized HPLC method on the same set of concentrations (0–90 ng/gm) of the mixed aflatoxin standard solution and spiked samples, the levels of aflatoxins predicted by the ATR-FTIR-PLSR when compared and verified with the values obtained by the HPLC, were not significantly different (p < 0.05) from the HPLC values. When both HPLC and ATR-FTIR-PLSR predicted concentrations were compared using fit regression (Fig. 5) to measure the correlation between the two methods, an excellent linear regression *R*^2^ value was obtained with a *p*-value of 0.001. The high *R*^2^ value obtained between the gold-standard HPLC method and ATR-FTIR-PLSR confirms the accuracy and precision of the new method as also obtained [18,50].

**Table 1.** PLSR model selection table.

Type of solvent	FTIR peak (cm^−^^1^)	Spectra region (cm^−^^1^)	Calibration fit		Cross-validation		*p*-value
			*R* ^2^	RMSEC	*R* ^2^	RMSEP	
100% ACN	2,970	2,980–2,961	0.9890	3.7214	0.9647	5.1435	0.001
	1,450	1,460–1,441	0.4776	19.7909	0.0475	26.7241	0.001
	1,035	1,063–1,001	0.9946	2.2957	0.9786	4.0034	0.001
	2,970+1,450 + 1,035	“3,000–2,941” “1,461–1,440” “1,062–1,000”	0.9950	1.9397	0.9934	2.2206	0.001
75% MeOH	2,970	3,000–2,941	0.9477	6.6201	0.9248	7.5073	0.001
	1,450	1,461–1,440	0.7075	16.1693	0.4611	20.1014	0.003
	1,035	1,062–1,000	0.9959	1.6948	0.9947	1.9846	0.001
	2,970+1,450 + 1,035	“3,000–2,941” “1,461–1,440” “1,062–1,000”	0.9956	1.7655	0.9939	2.1275	0.001

Where: RMSEP is the root mean square of the standard error of prediction and RMSEC is the root mean square of the standard error of calibration.

**Figure 3.**
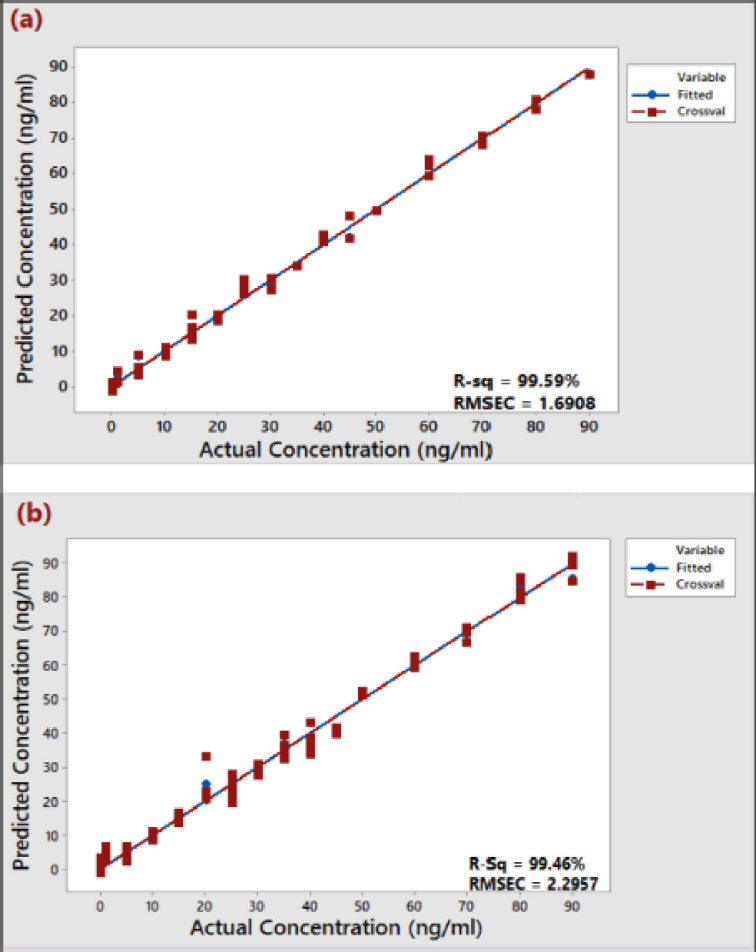
PLS response plot of ATR-FTIR predicted concentration versus actual concentration of total aflatoxins at the selected ATR-FTIR spectra region (1,062–1,000 cm^−1^) of the standard aflatoxins (0–90 ng/ml) prepared in (a) 75% MeOH and (b) ACN solvents.

**Figure 4.**
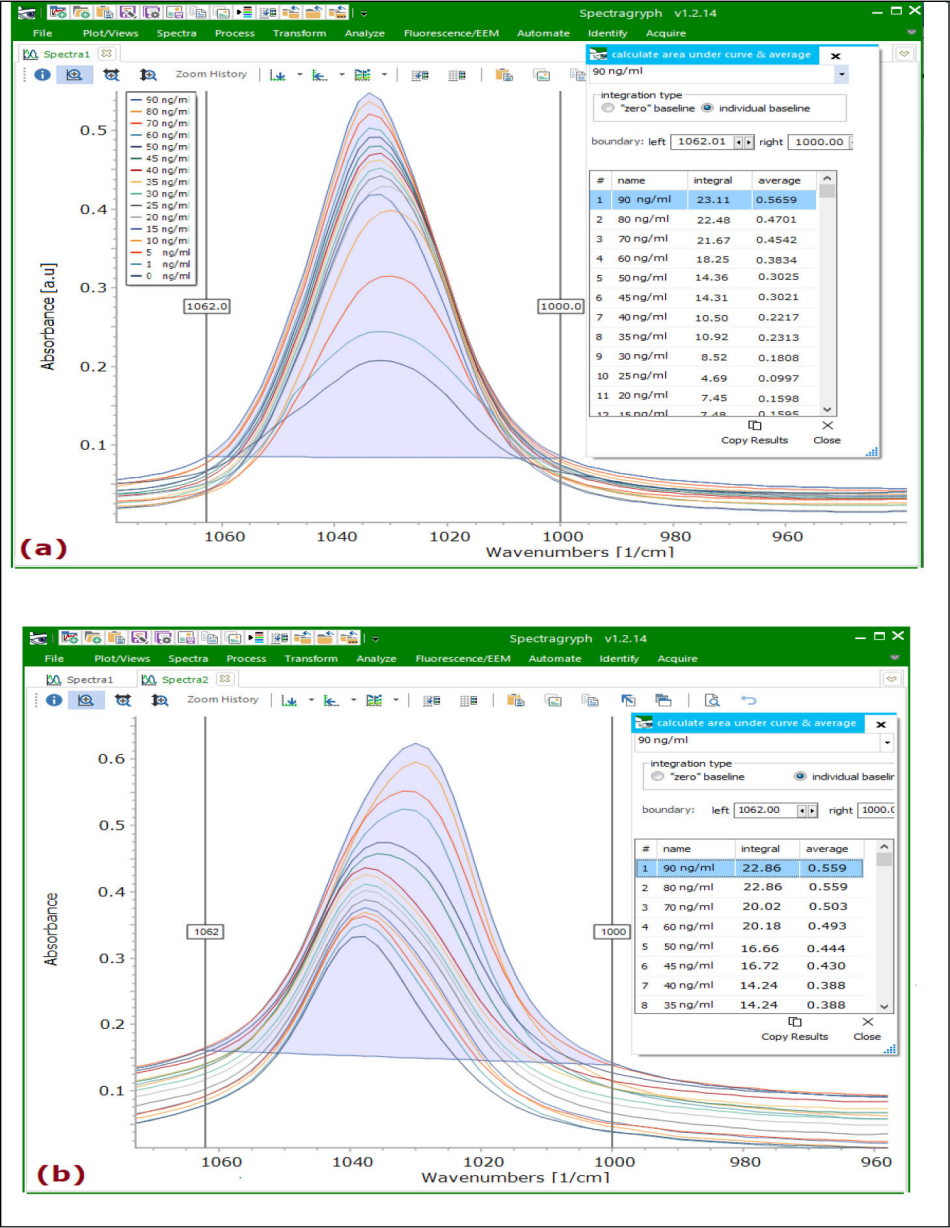
Spectragryph software interface showing the integration of the ATR-FTIR spectra of standard aflatoxins prepared in (a) 75% MeOH and (b) ACN solvents at a frequency region (1,062–1,000 cm^−1^).

The FTIR method’s performance was further compared with the HPLC method using Bland-Altman’s test [51] to determine their extent of agreement. The Bland-Altman plot (Fig. 6) showed good agreement between the two methods’ measurements at a 95% confidence level. This implies that the HPLC method is not significantly different from the proposed ATR-FTIR-PLSR method. So, the FTIR method can quickly figure out how much total aflatoxins are in chicken feed and food grains.

Overall, the proposed method showed high sensitivity (LoQ and LoD < 5.0 ng/gm), specificity (sample matrix does not affect the detection of aflatoxins at the selected spectra region), precision (CV, values between 0.97 and 1.72), and accuracy (% recovery was between 88% and 106%) in all the spiked chicken feeds and food grains samples.

### Determination of total aflatoxins in the commercial chicken feeds and food grains samples

The result of total aflatoxin levels in the 220 composted commercial chicken feeds and food grains determined using the validated ATR-FTIR-PLSR method is summarized in Table 3. The Aflatoxin levels in the Nigerian samples were statistically higher (p < 0.05) than those obtained in the Malaysian samples. This is not surprising because, unlike in Nigeria, where more than 80% of the food grains being sold in the markets come directly from the field/farmers without necessarily undergoing any aflatoxin screening process, most of the food grains used in Malaysia are imported products that are subjected to aflatoxin screening before being released to consumers.

A significant number of both the Nigerian chicken feeds (47.92%) and food grains (64.3%) analyzed were contaminated by aflatoxins at a range of 1.4–93.6 and 1.0–79.0 ng/gm, respectively, with 72% and 45% exceeding the maximum permissible level (20 ng/gm) of total aflatoxins in poultry feeds and foods imposed by the Nigerian government and some other countries such as Austria, Brazil, and the USA [52]. But the levels of aflatoxins in the samples tested for this study are lower than those found in similar studies in Nigeria [53–59] but higher than those of some other researchers [60–67].

**Table 2.** Relationship of the integral spectral peak areas and average absorbance responses with the respective aflatoxin concentration at 1,062–1,000 cm^−1^ ATR-FTIR spectral wavenumbers.

Type of solvent	Peak area (*Y*) and concentration (×)		Average absorbance (*Y*) and concentration (×)	
	Regression equation	*R* ^2^	Regression equation	*R* ^2^
75% MeOH	*Y* = 0.2151× + 4.8405	0.9372	*Y* = 0.0049× + 0.1	0.9064
ACN	*Y* = 0.1275 + 11.1450	0.8964	*Y* = 0.0028× + 0.3098	0.9283

Note: *p*-value in all the four regressions is less than the significant level (0.05).

**Figure 5.**
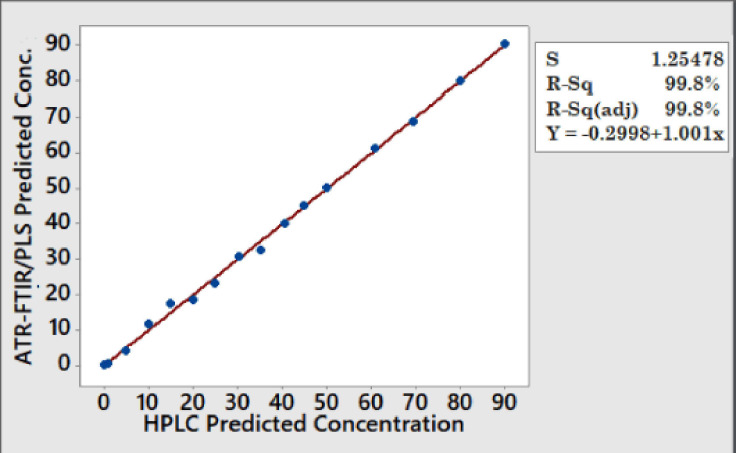
Fit regression between the ATR-FTIR-PLSR predicted concentration of aflatoxins and the HPLC predicted concentration.

**Figure 6.**
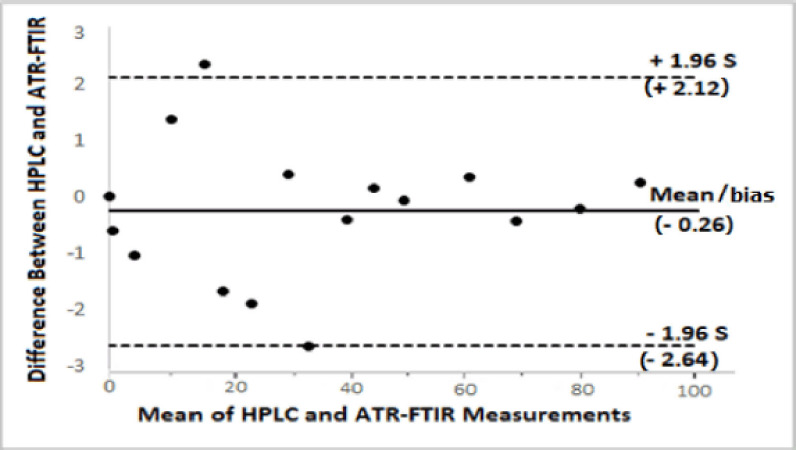
Bland-Altman‘s plot of the level of agreement between the HPLC and the ATR-FTIR measurements of aflatoxin concentrations (0–90 ng/ml) at a 95% confidence interval. Almost all the measurements fall within the acceptable level of +1.96 S to –1.96 S where S stands for the standard deviation of the difference between the two methods.

**Table 3.** Levels of aflatoxin contamination in the commercial food grains and poultry feeds.

Sample source	Sample type	*N*	*n*	Total aflatoxin concentration (ng/gm)		
				Mean ± SD	SE mean	Min - Max
Nigeria	Chicken feed	48	25	28.47 ± 21.4	4.3	1.0–79.0
	Rice	21	16	29.64 ± 22.89	5.7	5.1–81.8
	Wheat	21	14	46.36 ± 40.06	10.7	1.4–93.6
	Maize	21	13	28.38 ± 12.91	3.5	9.4–49.8
	Peanut	21	12	37.52 ± 37.42	10.8	1.5–87.3
Malaysia	Chicken feed	44	26	19.5 ± 11.72	0.7	7.2–53.2
	Rice	11	7	6.52 ± 3.83	1.4	4.9–11.9
	Wheat	11	5	28.30 ± 24.1	10.4	14.2–71.15
	Maize	11	3	15.54 ± 12.26	7.1	4.1–26.7
	Peanut	11	4	16.42 ± 6.75	3.4	8.6–24.8

Note: Values were obtained as the mean of triplicate. The letter *N* represents the total number of samples analyzed, *n* represents the total number of positive samples, SD is the standard deviation, SE is the standard error, and min and max are the minimum and maximum levels of aflatoxin contamination, respectively.

Similarly, 43.2% and 59.1% of the Malaysian chicken feeds and food grains analyzed in the present study were positive for aflatoxins at levels ranging from 4.1 to 71.15 ng/gm and 7.2 to 53.20 ng/gm, respectively (Table 3). Despite the high prevalence rate, most food grains contained aflatoxins below the Malaysian maximum acceptable level of 35 ng/gm for total aflatoxins in food grains. FAO [52] agreed with similar studies that obtained low levels of aflatoxins in Malaysian cereals and peanuts [33,68–73]. However, some researchers found higher levels of aflatoxins in Malaysian food grains than in the current study [69,73]. Conversely, 54% of the aflatoxin-positive chicken feed samples in the present study had aflatoxins above the acceptable European limit of 35 ng/gm [52]. However, the mean level of aflatoxins in the chicken feed is lower than that of the food grains. Overall, the levels of aflatoxin in this study show that both countries need to improve their current intervention and control plans for aflatoxins.

### Dietary exposure risk assessment of aflatoxins

Among the four most common aflatoxins (AFB1, AFB2, AFG1, and AFG2), out of about 20 naturally synthesized aflatoxins identified so far, AFB1 is the most toxic/carcinogenic and most detected fungal toxin (either singly or in combination with other aflatoxins) in foods and feeds. Research showed that other aflatoxins are not detected without AFB1 [74]. Hence, dietary exposure risks to aflatoxins (DERA) estimations for total aflatoxins are calculated based on the toxigenic potency of AFB1 in foods and feeds or its excretory form (AFM1) in the case of milk and urine exposure risk assessments. This study assessed dietary exposure risk and consequent risk of HCC in the exposed populations based on the levels of total aflatoxins in 128 composite samples of rice, wheat, maize, and peanuts from Nigeria and Malaysia (Table 4).

Where:

- Average food intake per person per day in Nigeria: maize = 60 gm [76], Peanut = 36.85 gm [77], Rice = 101.37 gm [78], and Wheat = 54.79 gm [79]
- Average daily food intake in Malaysia per person: Peanut = 4.47 gm[80], Rice = 225.5 gm[81], and Cereals = 23.8 gm[82]*.*
- Average person’s body weight in Nigeria = 60.0K gm [75], in Malaysia = 62.65 kg [83]
- Average estimated potency of cancer for peoples in Nigeria is 0.0825 cancers/100,000 peoples/year [75], in Malaysia is 0.025 cancers/100,000 peoples/year [83]
- Average incidence of liver cancer for peoples in Nigeria is 7.13/100,000 population/year [84], in Malaysia = 4.9/100,000 population/year [85].

The levels of total aflatoxins obtained in the Nigerian samples signify very high dietary exposure risk to aflatoxins ranging from 0.92 to 138.2 ng/kg BW/day (mean range = 23.04–50.08 ng/kg BW/day), which could lead to an estimated 1.07%–159.91% of HCC/100,000 people/year with a mean range of 26.6%–57.94% of HCC/100,000 people/year in the exposed population. Consumption of rice could pose the greatest risk of aflatoxin exposure (50.08 ng/kg BW/day), resulting in a 57.94% yearly incidence of HCC/100,000 people in the exposed population. In decreasing order, wheat, maize, and peanuts could account for an estimated 48.08%, 32.84%, and 26.66% of HCC/100,000 people in the exposed population per year, respectively.

Generally, studies show that Africans are being exposed to high dietary aflatoxins exceeding 100 ng/kg BW/day compared to most developed countries, where the levels of exposure are as low as 1.0 ng/kg BW/day or less [86]. A recent review on dietary aflatoxin exposure in Nigeria revealed that consumption of maize, rice, peanut, and wheat could be attributed to a very high risk of dietary aflatoxin exposure ranging from 1.7 × 10^−4^ ng/kg BW/day to 9. 88 × 10^4^ ng/kg BW/day, 1.28 to 628.49 ng/kg BW/day, 0.55 to 396.75 ng/kg BW/day, and 1.53 to 18.75 ng/kg BW/day, respectively; which could account for an estimated percentage incidence of HCC/100,000 population/year of 0.0024%–708.13% with a mean range of 160.60%–176.44% between 1998 and 2008 and 0.0046%–45,602% with a mean range of 84.03%–1,052.50% between 2009 and 2018 in the country [87]. The findings of this study support the findings of Atanda et al. [88]. According to his interview with certain doctors from special hospitals in Nigeria, several post-mortem studies on individuals who died of liver cancer connected the cause of death to aflatoxins. Some other doctors reported that multiple cases of aflatoxicosis and other mycotoxicoses have been occurring in Nigeria, most of which have not been published or reported [88].

Conversely, the mean total aflatoxins obtained in the Malaysian samples indicate a very lower DERA than in Nigeria. The dietary aflatoxin exposure risk signified by the Malaysian samples ranges from 1.56 to 27.03 ng/kg BW/day (mean range = 5.90–11.02 ng/kg BW/day), which could lead to an estimated 0.79%–13.79% of HCC/100,000 people/year with a mean range of 3.01%–5.62% incidence of HCC/100,000 people/year in the exposed population. Like Nigeria, rice consumption in Malaysia could pose the highest risk of aflatoxin exposure (11.02 ng/kg BW/day), which might result in a 5.62% yearly incidence of HCC/100,000 people in the exposed population. This is followed by wheat, peanuts, and maize, which could account for an estimated 5.49%, 5.15%, and 3.01% of HCC/100,000 people in the exposed population per year. The percentage of HCC incident rates (0.79%–13.79%) obtained in this study is similar to the previously reported % incident rates of 5.5% in 2010 [89], 0.61%–14.9% in 2011 [90], 13.5% in 2012 [91], and 12.4%–17.3% in 2012 [83] in Malaysia. However, incident rates were reported at a higher percentage in 2010 (91%–857%) [92] and 2013 (14.7%–29.6%) [93].

**Table 4.** Estimated dietary exposure to aflatoxins and attributable cases of HCC in Nigeria and Malaysia.

Country	Type of sample analysed	Variables	Aflatoxins levels (ng/gm)	Calculated DERA (ng/kgbw/day)	Calculated HCC per 100,000/year	% Incidence of the HCC per 100,000
Nigeria	Rice	Mean	29.64	50.08	4.13	57.94
		Min	5.1	8.62	0.71	9.97
		Max	81.8	138.20	11.40	159.91
	Wheat	Mean	46.36	42.33	3.49	48.98
		Min	1.4	1.28	0.11	1.48
		Max	93.6	57.49	4.74	66.52
	Maize	Mean	28.38	28.38	2.34	32.84
		Min	9.4	9.40	0.78	10.88
		Max	49.8	49.80	4.11	57.62
	Peanut	Mean	37.52	23.04	1.90	26.66
		Min	1.5	0.92	0.08	1.07
		Max	87.3	53.62	4.42	62.04
Malaysia	Rice	Mean	6.52	11.02	0.28	5.62
		Min	4.9	8.28	0.21	4.22
		Max	11.9	20.11	0.50	10.26
	Wheat	Mean	28.3	10.75	0.27	5.49
		Min	14.4	5.47	0.14	2.79
		Max	71.15	27.03	0.68	13.79
	Maize	Mean	15.54	5.90	0.15	3.01
		Min	4.1	1.56	0.04	0.79
		Max	26.7	10.14	0.25	5.18
	Peanut	Mean	16.42	10.08	0.25	5.15
		Min	8.6	5.28	0.13	2.69
		Max	24.8	15.23	0.38	7.77

Note:

– Min and Max are the minimum and maximum levels of aflatoxins in the samples, respectively.

– Calculated DERA (ng/kg BW/day) was computed as:

– Level of aflatoxins in food (ng/g) × Daily food intake per person (g/day) / Average bogy weight of an adult in the population (kgbw)(2) [75]

– Calculated HCC per 100,000/year was computed as:

– Estimated DERA (ng/KgBw/day) × Average cancer potency in the population (3) 75

– % Incidence of the HCC per 100,000 was computed as:

– Attributable HCC per 100,000/year / Average liver cancer incidence in the population × 100 (4) 75

## Conclusion

The proposed PLSR integrated ATR-FTIR spectroscopy model at a frequency region of 1,062–1,000 cm^−1^ has demonstrated high potential for rapid qualitative and quantitative detection of total aflatoxins in chicken feeds and food grains over the standard chromatographic methods. However, more studies are needed to evaluate further and validate the method‘s performance using various other extraction methods/solvents/clean-up techniques, as well as the applicability of the method to real-time analysis of raw samples that have not been treated with extraction solvents. Furthermore, the aflatoxin levels obtained in the commercial samples signify a greater dietary exposure risk to aflatoxins and consequent HCC risk in the exposed populations (Malaysia and Nigeria). Hence, there is a need to strengthen the control, prevention, and intervention methods to minimize the dietary exposure risk to aflatoxins associated with consuming commercial/marketed foods in both countries. Consequently, the level of aflatoxins in chicken feeds calls for studies assessing the levels of aflatoxins in commercial poultry products such as meat and eggs.

## Figures and Tables

**Figure 1S.**
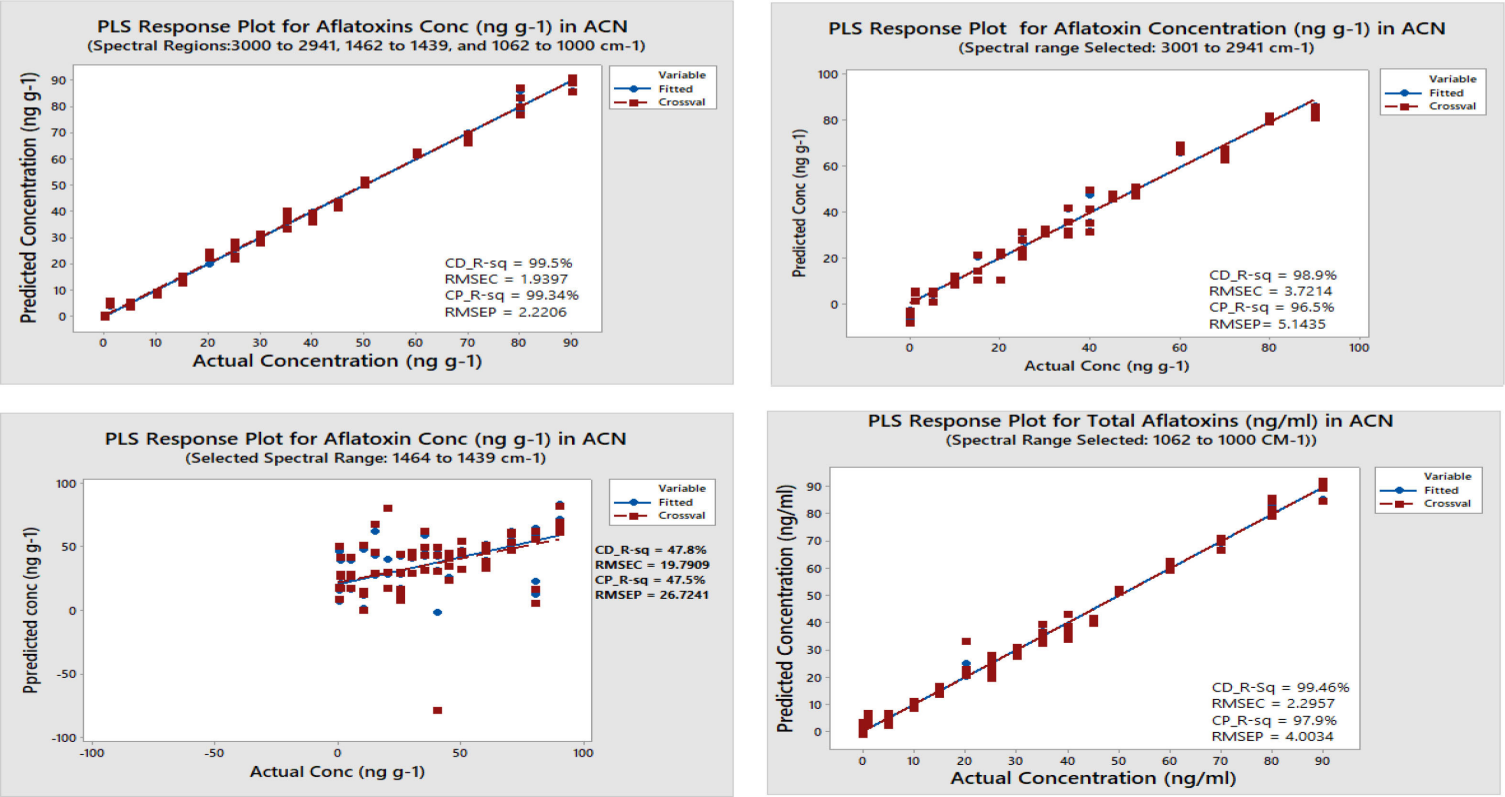
(Model selection plots in ACN) PLSR response plots of aflatoxins in ACN solvent showing the ATR-FTIR predicted concentration versus actual concentration of total aflatoxins at the various ATR-FTIR spectra regions used for PLSR model selection.

**Figure 2S.**
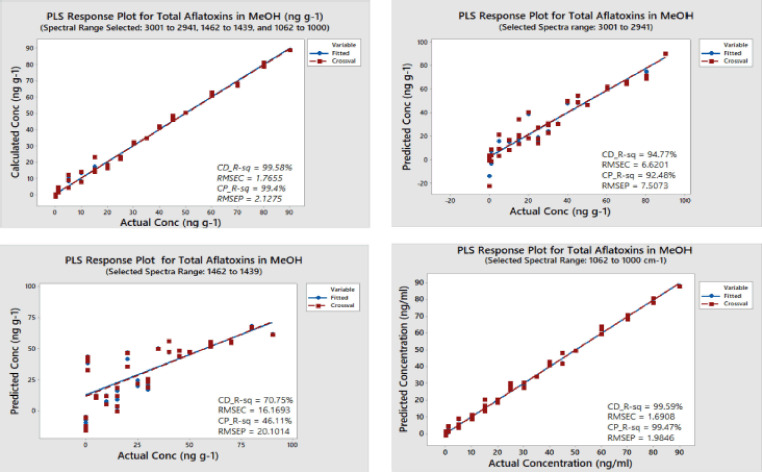
(Model selection plots in MeOH) PLS response plots of aflatoxins in methanol solvent showing the ATR-FTIR predicted concentration versus actual concentration of total aflatoxins at the selected ATR-FTIR spectra regions used for PLSR model selection.

**Figure 3S.**
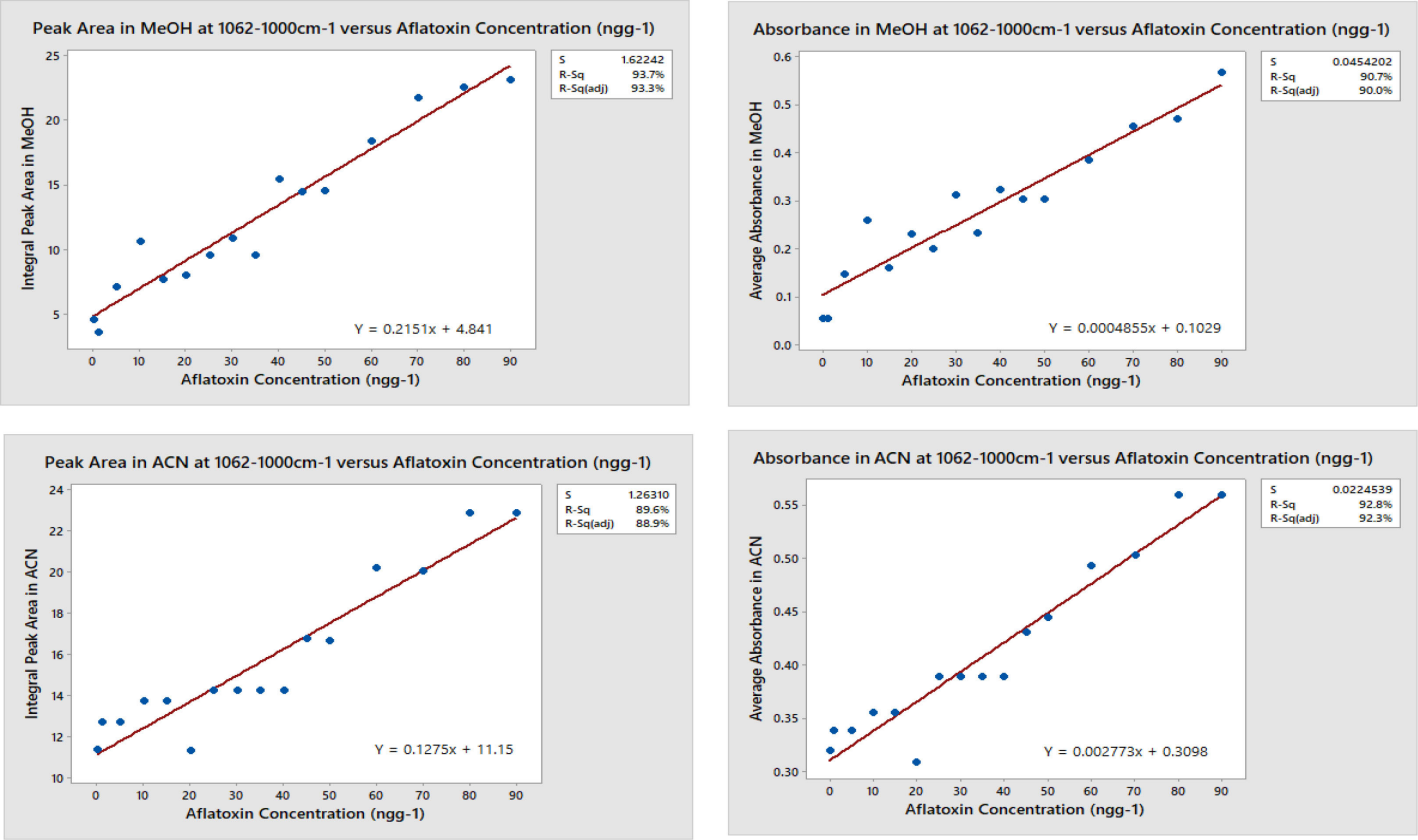
Fit regression plots of spectral peak areas and absorbances versus respective aflatoxins’ concentrations in both methanol solvent and ACN at the best frequency region selected from the Figures S1 and S2 for the PLSR.

## List of Abbreviations

°C, Degree celsius; >, Greater than; <, Less than; µl, microliter; kg BW/day, Kilogram body weight per day; ng/ml, Nanogram per milliliter; ng/gm, Nanogram per gram; nm, Nanometer; ACN, Acetonitrile; AFB1, Aflatoxin B1; AFG1, Aflatoxin G1; AFB2, Aflatoxin B2; AFG2, Aflatoxin G2; ATR-FTIR, Attenuated total reflectance–Fourier transformed infrared spectroscopy; CV, Coefficient of variation; DERA, dietary exposure risks to aflatoxins; ELISA, Enzyme-linked immunosorbent assay; HCC, hepatocellular carcinoma; HPLC, High-performance liquid chromatography; MeOH, methanol; MIR, Mid-infrared; RMSEC, Root mean square error of calibration; RMSEP, Root mean square error of prediction; PLSR, Partial least square regression.
